# Molecular approach to confirm traditional identification of *Radopholus similis* sampled in Tanzania

**DOI:** 10.21307/jofnem-2020-020

**Published:** 2020-03-17

**Authors:** Doreen M. Mgonja, Gladness E. Temu, Joseph C. Ndunguru, Magreth F. Mziray, Sylvester L. Lyantagaye, Nessie D. Luambano

**Affiliations:** 1 College of Natural and Applied Sciences, University of Dar es Salaam, P.O. Box 35091, Dar es Salaam, Tanzania; 2 Tanzania Agricultural Research Institute, Mikocheni, P.O. Box 6226, Dar es Salaam, Tanzania; 3 Tanzania Agricultural Research Institute, Kibaha, P.O. Box 30031, Kibaha, Tanzania; 4 Mbeya College of Health and Allied Sciences, University of Dar es Salaam, P.O. Box 608, Mbeya, Tanzania

**Keywords:** Banana, Burrowing nematodes, Genetic variation, *Musa* spp., Pest management, Plant-parasitic nematodes, Taxonomy.

## Abstract

Banana (*Musa* spp. L.) is an important staple food and cash crop for about 30% of the population in Tanzania; however, the burrowing plant-parasitic nematode *Radopholus similis* causes black head disease and toppling in banana plants, which results in yield losses. We collected and identified 80 specimens of *R. similis* from four agro-ecological zones in Tanzania using morphological characters. We then used universal and specific *R. similis* primers to amplify the small subunit, internal transcribed spacer and large subunit of ribosomal DNA regions of these specimens. The amplicons were subsequently sequenced and analyzed using Bayesian inference. We identified two major clades, one that comprised all *R. similis* sequences derived from this study and another that included *R. similis* and *Radopholus* spp. sequences obtained from GenBank, indicating the separation of this species from congeneric sequences. Our findings provide a useful, simple and rapid method for identifying burrowing nematodes. This outcome could contribute to the development of permanent, integrated pest management strategies for the control of *R. similis* in banana and other crops in order to reduce associated yield losses in Tanzania. To our knowledge, this is the first study of nematodes to use combined morphological and molecular methods for the identification of *R. similis* in Tanzania.

Banana (*Musa* spp. L.) is a key food crop in rural and urban areas of the humid tropics, with an annual global production of up to 100mn tons (FAO, 2015). In East Africa, banana is widely consumed and provides approximately 10% of the calorific intake for more than 70mn people (Kilimo Trust, 2012). In Tanzania, in particular, it is a staple food and cash crop for more than 30% of the total population (Nkuba, 2007).

Plant-parasitic nematodes (PPN) are the principal pests of banana in Tanzania, adversely affecting banana production by causing up to 50% yield losses (Coyne, 2009). However, the effective management of *R. similis* in banana crops is problematic, because the limited control strategies available at present tend not to be used by small-scale farmers as they involve the use of hot water and expensive nematicides, many of which are environmentally hazardous (Van, 2013). Thus, burrowing nematode species need rapid and accurate identification in order to allow development of alternative and permanent sustainable management strategies specific to *R. similis* (Kaplan et al., 2000). This would contribute to achieving the estimated annual banana production potential of 10 Mt/ha in Tanzania (Kilimo Trust, 2012). Morphological features have been used in the identification of *R. similis* in Tanzania (Speijer and De Waele, 2001; Coyne, 2009). More recent research suggests that the use of only morphological features increases the risk of misidentification (Handoo et al., 2016; Wang et al., 2016). Thus, additional identification methods, particularly molecular-based identification, which requires a small amount of nucleic acid extracted from a single individual, will allow more accurate nematode identification, regardless of developmental stage (Handoo et al., 2016). The present study aimed to characterize *R. similis* using a combined morphological and morphometric approach and to confirm its identity using rDNA sequencing.

## Materials and methods

### Nematode populations

We collected 314 root samples from 104 smallholder farms distributed across four agro-ecological zones of Tanzania, comprising three on the mainland (Kagera region in the Lake zone, Mbeya and Ruvuma regions in the Southern Highlands zone, and Kilimanjaro and Arusha regions in the Northern zone) and one on the Zanzibar islands of Unguja and Pemba. Out of 24 fields, 10 were surveyed in each region. The variation on the number of fields surveyed from one region to another was due to the availability of banana fields. Three samples of 10 to 15-cm lengths, each weighing approximately 20 g, of banana roots were collected randomly from each banana field as described by Luambano et al. (2019).

### Nematode extraction and identification

Nematode extraction was done using a modified Baermann’s method as explained by Coyne et al. (2007), by which 5 g of banana roots were extracted from each sample. The extracted material was subsequently incubated for 24 hr, after which a dissecting microscope (Leica MZ 9.5, Heerbrugg, Switzerland) was used to observe nematodes from the extracted material.

### Morphological and morphometric characterization

From the 314 banana root samples, more than 20 nematodes were extracted from each zone. In total, 20 (10 males and 10 females) were picked for morphological and morphometric characterization under a dissecting microscope (Leica MZ 9.5) based on characters associated with *R. similis* including: presence of three to four lip annuli; long stylet with rounded and flattened basal knobs; elongated tail with pointed terminus and vulva positioned slightly below mid body (54-55%) in females; presence of a strongly offset and knob-shaped head; degenerated pharynx and stylet with very small stylet knobs in males (Siddiqi, 2000). Extracted nematodes were placed on a glass slide with 10 to 20 μl of sddH_2_O, and specimens were then further observed under a compound microscope (Leica 2500, Leica Microsystems CMS GmbH, Wetzler, Germany) at 20× magnification to confirm initial identification based on morphology. Nematodes were identified from digital photographs (40× magnification) and under 100× magnification with oil immersion. They were measured according to parameters described by Handoo et al. (2016).

### Molecular characterization

PCR amplification of small subunit (SSU), internal transcribed spacers 1 and 2 (ITS1 and ITS2) and large subunit (LSU) of rDNA regions of nematodes collected from the four agro-ecological zones were used for molecular characterization of *R. similis*.

The DNA extraction was carried out according to the protocol described by Ye et al. (2015). A single adult *R. similis* nematode was removed from the glass microscope slide previously used for morphological analysis thus, made a total of 20 DNA samples from each zone.

### PCR amplification, cleaning and sequencing

The extracted DNAs were used for PCR amplification, using universal primers 18S965/18S1573R GGCGATCAGATACCGCCCTAGTT/TACAAAGGGCAGGGACGTAAT (Mullin et al., 2005) and rDNA2/rDNA 1.58 S TTGATTACGTTCCCTGCCCTTT/ACGAGCCGAGTGATCCACCG (Vrain et al., 1992; Cherry et al., 1997) to amplify the SSU & ITS1 rDNA regions, respectively, for the preliminary identification of *R. similis*. Additional species-specific primers were designed including; primers RD1f/RD1r (ACTGAGCCGATTCGAGAAATC/ATGATTTGGAAAAGCTGCCAATTT); RS3ITSF/RS3ITSR (CTGTGAGTCGTGGAGCAGTT/ATGATTTGGAAAAGCTGCCAAT) and RS4ITSF/RS4ITSR (TGTAGTCCATGTCCGTGGC/TGATTTGGAAAAGCTGCCAATTT) which amplify the ITS1 & ITS2 and primers RS8LSUF/RS8LSUR (AGGACGTGAAACCGGTGAGG/TATACCCAAGTCAGACGATCG) and RS6LSUF/RS6LSUR (CTGGCGTATCTAGCCTGCAT/TTTACACCGAGGATTGGCGT), which target the LSU rDNA regions for confirmation of morphological identification. These primers were designed using the Primer3 and BLAST tool from the NCBI site (https://www.ncbi.nlm.nih.gov/tools/primer-blast/) and synthesized by the Bioneer Corporation, Daejeon, Republic of South Korea. The specificity of each primer to amplify target nematode species was evaluated using pretested DNA for *R. similis*, *P. goodeyi* and *P. coffeae*, which were obtained by PCR amplification using universal primers 18S965/18S1573R and rDNA2/rDNA1.58 S and species were determined by sequencing.

The PCR amplification using the designed primers was done as described by Ye et al. (2015). The thermocycler conditions for amplification comprised initial denaturation at 94°C for 3 min, followed by 35 cycles of denaturation at 94°C for 30 sec, annealing for 45 sec at 55°C for ITS primers and 49°C for LSU primers, extension at 72°C for 1 min, and a final extension at 72°C for 10 min. Amplicons were analyzed on 1% agarose gel electrophoresis and cleaned using ExoSap-IT (Affymetrix Inc., Santa Clara, CA, USA) before they were submitted for direct Sanger sequencing to the Genomic Sciences Laboratory (North Carolina State University, Raleigh, NC, USA).

### Sequencing analysis and phylogeny

Original *R. similis* sequences, which were deposited in GenBank, were compared with other nematode species sequences available in GenBank through NCBI BLASTN homology searches. Multiple sequence comparison by log-expectation was done using Geneious version 11.0 software (Kearse et al., 2012). Bayesian inference was used to construct phylogenetic trees in the Geneious version 11.0 software. The model selected was the best fit for the SSU plus ITS1, ITS1 & ITS1 and LSU data set, the HKY + G + I (Hasegawa et al., 1985), and *Helicotylenchus* spp. were selected as outgroups for the data sets.

### Statistical analysis

Differences in morphological parameters among the regions were tested using analysis of variance in GENSTAT (14th edition, VSN International Ltd, Hemel Hempstead, UK) to compare the means using the least significance difference test with a statistical threshold of *p* < 0.005 (Roy et al., 2018).

## Results

### Morphological and morphometrics

**Figure 1:**
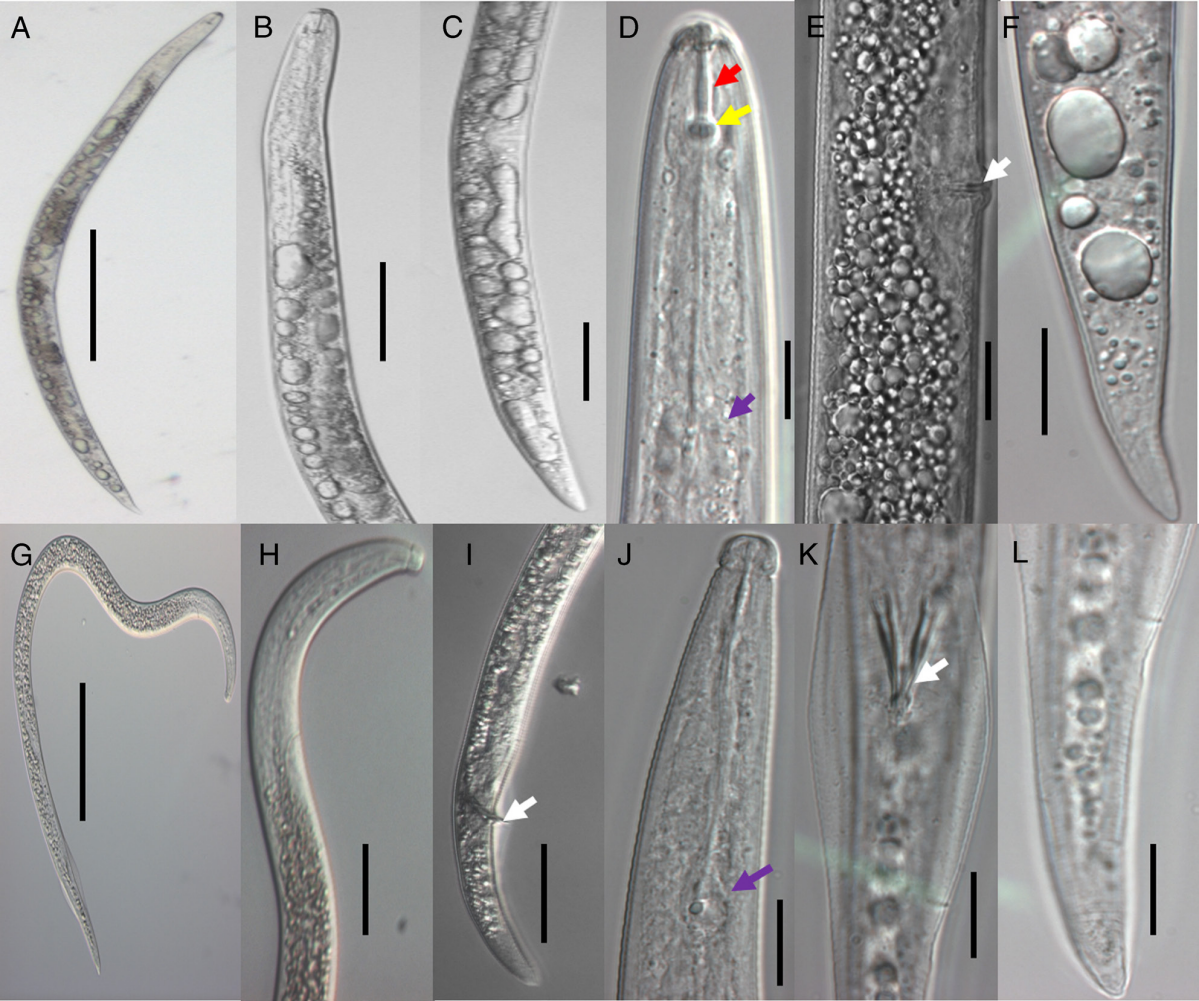
Micrographs of *Radophilis similis* collected from Tanzania. Representative anatomy shown for males (A-F) and females (G-L): (A, G) Entire body structure; (B, H) pharyngeal region; (C, I) posterior region; (D, J) anterior region (stylet, stylet knobs, and median bulb shown by red, yellow, and purple arrows, respectively); (E, K) posterior region (vulva and spicule shown by white arrows); and (F, L) tail region. Scale bars: 50 μm (A-G) and 25 μm (B-F, H-L).

*Radopholus similis* was detected in all four zones and there were few morphometric differences among the zones in either female or males. In females, the body was straight or slightly curved ventrally; the head was low, rounded and continuous with three to four lip annuli; the long stylet was well developed with rounded and flattened basal knobs; excretory pores were present at the esophago-intestinal junction; the tail was elongated with a pointed terminus; the pharyngeal gland dorsally overlapped the intestine; and the vulva was positioned post equatorial, with approximately 54 to 55% of body length at the anterior (Fig. 1A-F). In males, the body was slender and ventrally curved; the pharynx and stylet degenerated were with very small stylet knobs and an indistinct median bulb; head was strongly offset and knob-shaped; spicules were strong and long (about 18-22 μm) with pointed distal ends (Fig. 1G-L).

The lengths of female and male *R. similis* were within the expected lengths of 500 to 600 and 440 to 685 μm, respectively. Longest average body lengths of females and males were recorded from Zanzibar (581.0 ± 11.4 and 570.5 ± 12.2 μm, respectively), and the shortest average body lengths was recorded in the Lake zone (520.5 ± 11.4 and 486.3 ± 12.2 μm, respectively). The *R. similis* females from Zanzibar also had the greatest average body width (18.8 ± 3.0 μm) compared to females from other zones. Additionally, the esophagus length (OL) of both sexes was within the standard range and the greatest average OL was from the Northern zone with 91.8 ± 2.6 μm (females) and 80.1 ± 2.2 μm (males). Stylet length in females and males from all zones were outside the standard measurements (Table 1) except in males from the Lake and Northern zones.

**Table 1.** Morphometrics (μm) of adult *R. similis* female and male populations collected from the agro-ecological zones sampled. Data are means ± SD and standard range (Roy et al., 2018; Elbadri et al., 1999) in parentheses.

	Female populations				Male populations			
Parameter^a^	Lake zone	Northern zone	Southern Highlands zone	Zanzibar islands	Lake zone	Northern zone	Southern Highlands zone	Zanzibar islands
L	520.5 ± 11.4	564.1 ± 11.4	554.3 ± 11.4	581.0 ± 11.4	486.3 ± 12.2	566.1 ± 12.2	542.8 ± 12.2	570.5 ± 12.2
	(500 – 600)	(500–600)	(500 – 600)	(500 – 600)	(440 – 685)	(440–685)	(440 – 685)	(440 – 685)
a	28.5 ± 5.1	28.5 ± 5.1	27.3 ± 3.9	30.3 ± 2.7	29.5 ± 3.8	31.8 ± 5.2	33.1 ± 3.6	33.1 ± 3.6
	(20 – 34.0)	(20 – 34.0)	(20 – 34.0)	(20 – 34.0)	(24.5 – 42.5)	(24.5 – 42.5)	(24.5 – 42.5)	(24.5 – 42.5)
b	6.3 ± 1.1	4.93 ± 0.7	5.79 ± 0.7	5.6 ± 0.8	5.2 ± 0.3	5.3 ± 0.5	5.4 ± 0.3	5.1 ± 0.2
	(4.2 – 6.6)	(4.2 – 6.6)	(4.2 – 6.6)	(4.2 – 6.6)	(4.1 – 5.6)	(4.1 – 5.6)	(4.1 – 5.6)	(4.1 – 5.6)
b'	5.1 ± 0.5	4.2 ± 0.6	4.9 ± 0.5	4.8 ± 0.6	5.4 ± 0.5	5.4 ± 0.7	5.5 ± 0.8	5.9 ± 0.7
	(3.3 – 5.7)	(3.3 – 5.7)	(3.3 – 5.7)	(3.3 – 5.7)	(3.7 – 6.8)	(3.7 – 6.8)	(3.7 – 6.8)	(3.7 – 6.8)
c	8.2 ± 1.0	7.9 ± 0.5	7.5 ± 0.5	8.4 ± 1.2	6.8 ± 0.3	6.8 ± 0.6	7.3 ± 0.1	6.9 ± 0.6
	(6.8 – 12.2)	(6.8 – 12.2)	(6.8 – 12.2)	(6.8 – 12.2)	(6.3 – 10.3)	(6.3 – 10.3)	(6.3 – 10.3)	(6.3 – 10.3)
c'	4.4 ± 0.7	4.2 ± 1.0	5.12 ± 0.8	4.8 ± 0.5	5.8 ± 0.7	5.7 ± 1.0	5.8 ± 1.1	6.4 ± 0.7
	(2.8 – 6.2)	(2.8 – 6.2)	(2.8 – 6.2)	(2.8 – 6.2)	(3.7 – 6.9)	(3.7 – 6.9)	(3.7 – 6.9)	(3.7 – 6.9)
Sl	10.8 ± 1.1	10.6 ± 1.1	10.7 ± 1.1	10.4 ± 1.1	10.3 ± 2.2	10.0 ± 2.0	9.8 ± 2.2	8.7 ± 2.2
	(14 – 18)	(14 – 18)	(14 – 18)	(14 – 18)	(8 – 13)	(8 – 13)	(8 – 13)	(8 – 13)
V	51 ± 0.9	55.5 ± 0.9	55.4 ± 0.8	58.2 ± 0.7	131.7 ± 2.1	144.0 ± 2.1	131.6 ± 2.2	131.7 ± 2.1
	(50.7 – 59)	(50.7 – 59)	(50.7 – 59)	(50.7 – 59)	(128 – 250)	(128 – 250)	(128 – 250)	(128 – 250)
W	16.0 ± 3.0	18.0 ± 3.0	17.6 ± 3.0	18.8 ± 3.0	14.5 ± 2.0	16.1 ± 2.1	14.7 ± 2.1	14. 7 ± 2.3
	(13 – 21)	(13 – 21)	(13 – 21)	(13 – 21)	(14 – 18)	(14 – 18)	(14 – 18)	(14 – 18)
t	64.7 ± 2.0	73.3 ± 2.3	75.2 ± 2.0	72.3 ± 2.6	69.2 ± 2.0	81.8 ± 1.9	72.0 ± 2.1	80.5 ± 2.0
	(52 – 100)	(52 – 100)	(52 – 100)	(52 – 100)	(60 – 90)	(60 – 90)	(60 – 90)	(60 – 90)
OL	72.5 ± 2.6	91.8 ± 2.6	82.7 ± 3.1	84.8 ± 3.6	63.8 ± 2.1	80.1 ± 2.2	76.4 ± 2.2	72.5 ± 2.0
	(69 – 99)	(69 – 99)	(69 – 99)	(69 – 99)	(62 – 123)	(62 – 123)	(62 – 123)	(62 – 123)
DGO	3.0 ± 0.4	3.5 ± 0.5	3.2 ± 0.5	2.6 ± 0.5	1.8 ± 0.1	2.4 ± 0.5	2.6 ± 0.3	1.5 ± 0.3
	(2.0 – 5.5)	(2.0 – 5.5)	(2.0 – 5.5)	(2.0 – 5.5)	(1.5–3.0)	(1.5 – 3.0)	(1.5 – 3.0)	(1.5 – 3.0)

Note: *Abbreviations are defined in Siddiqi, (2000).

### Molecular characterization, sequencing and phylogenetic analysis

PCR amplification of *R. similis* using universal primer pairs targeting the SSU & ITS1 rDNA region produced fragments with expected sizes of 490 and 950 bp, respectively. In addition, PCR amplification using primer pairs targeting the ITS1 & ITS2 regions and the LSU rDNA region also produced fragments with expected sizes ranging between 308 and 750 bp.

**Figure 2:**
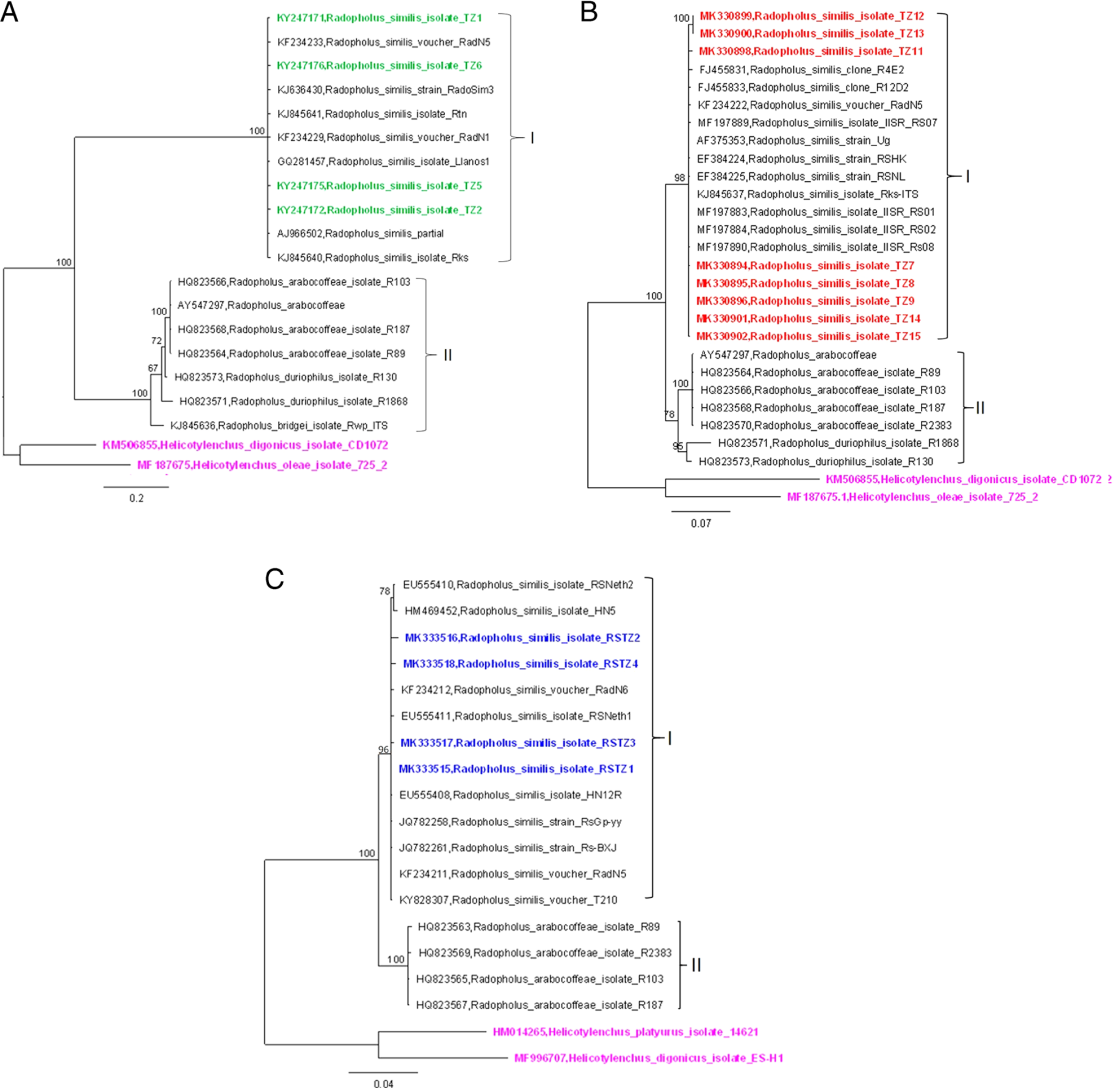
Bayesian consensus trees inferred for *Radophilis similis*. (A) Tree inferred from SSU & ITS1 rRNA. (B) Tree inferred from ITS1 and ITS2 rRNA. (C) Tree inferred from LSU rRNA. Posterior probability values >50% are shown. Sequences original to this study are labeled green, red, and blue for trees in (A), (B), and (C) respectively; sequences labeled pink represent outgroup species.

A BLASTN search of the SSU & ITS1 rDNA sequence from four populations of *R. similis* isolated in this study (KY247171, KY247172, KY247175 and KY247176) revealed high matches (99%) with previously published SSU & ITS1 rDNA sequences of *R. similis* (KF234233, AJ966502, GQ281457 and KJ636430, respectively). Our phylogenetic analysis, inferred from *R. similis* 18 S & ITS1 sequence (718 nt), revealed two distinct clades. The first comprised all *R. similis* isolates from Tanzania and *R. similis* isolates from other geographical regions (Fig. 2A). The second clade comprised members from *R. bridgei*, *R. duriophilus* and *R. arabocoffeae* isolates from elsewhere.

A BLASTN search of the ITS1 and ITS2 sequences of *R. similis* (FJ455831, MF197890 and AY547297) from GenBank revealed high similarity (99% identity) with the *R. similis* ITS1 and ITS2 sequences (MK330894-MK330896, MK330898, and MK330899-MK330902) found in this study. Phylogenetic relationships of the *R. similis* ITS1 and ITS2 sequences (610 nt) showed close similarity with previously published ITS1 and ITS2 sequences of *R. similis*. Two clades were obtained: “clade I” comprised of *R. similis* isolated from the present and previously published studies, while “clade II” comprised *R. arabocoffeae* and *R. duriophilus* (Fig. 2B).

Furthermore, a BLASTN search of four populations of *R. similis* 28 S rDNA sequences (MK333515-MK333518) isolated in this study showed a 99.3% matching to previously published rDNA sequences (EU555408, EU555411, and KF234212) and lower matches to other members of the *Radopholus* genus. Phylogenetic analysis of *R. similis* 28 S rDNA sequences (670 nt) identified two clades: “clade I,” which contained *R. similis* isolated from the present and previously published studies, and “clade II,” which contained *R. arabocoffeae* (Fig. 2C).

Assessment of *R. similis* isolates using rDNA sequences (18 S & ITS1, ITS1 & ITS2 and LSU rDNA regions) grouped all *R. similis* into one monophyletic group with respect to other species of *Radopholus* (Fig. 2A-C).

## Discussion

Identification and characterization of *R. similis* showed that this nematode species was present in banana crops in all agro-ecological zones included in this study, making it a potential threat to banana production in Tanzania. Morphological and morphometric characterization of *R. similis* generally concurred with descriptions and measurements reported in other studies (Ryss and Wouts, 1992; Elbadri et al., 1999; Murukesan et al., 2005; Roy et al., 2018). Most *R. similis* specimens from Zanzibar had greater total body length than those from the mainland. However, we found that male and female stylet lengths fell outside of standard diagnostic values (Ryss and Wouts, 1992; Elbadri et al., 1999), possibly due to variations in environmental conditions that drive morphological adaptation. We also found that males had slender bodies compared to females and degenerated stylets in all zones, reflecting the lower level of damage that males inflict as reported by Ryss and Wouts (1992) and Roy et al. (2018).

Molecular identification and phylogenetic analysis showed separate clustering of *R. similis* from other *Radopholus* species. The separation of *R. similis* from other *Radopholus* species in this study shows that the primers used are sufficient to amplify *R. similis* and they are able to distinguish the species from other *Radopholus* species as well as other PPNs. These results confirm the efficiency and accuracy of PCR-based methods in distinguishing congeneric nematodes.

The rDNA sequences, including LSU (28 S), ITS, and SSU (18 S), are important for nematode characterization because they collectively distinguish both distantly related from closely related species and between and within closely related species (Kaplan et al., 2000; Al-Banna et al., 2004; Zeng et al., 2015). Further, our findings agree with those of other studies on the importance of using a combined morphological and molecular approach for nematode species identification (Troccoli et al., 2008; Mahran et al., 2010). The close similarity among *R. similis* in this study and previously published sequences for *R. similis* confirmed its presence in Tanzania, specifically in addition to other locales in East Africa (Price, 2006). Our results show that *R. similis* was prevalent in Tanzanian banana plantations that were separated by >1,000 km across three agro-ecological zones on mainland Tanzania and one on the Zanzibar archipelago. This wide distribution suggests that *R. similis* may have been spread inadvertently through the movement of infested banana planting material from one area of the country to another.

To our knowledge, the present study is the first report of molecular evidence supporting the identification and occurrence of *R. similis* in Tanzania. Our findings provide a useful, simple and rapid methodology for identifying burrowing nematodes. This methodology could play an important role in the development of permanent integrated pest management strategies to mitigate the effects of *R. similis* in the cultivation of banana and other crops. This work also provides a basis for further nematode research, which should include a comprehensive survey of all major banana-producing regions to determine PPN distribution, for example as presented by Luambano et al. (2017), aggressiveness, and diversity of PPN species that affect banana production in Tanzania, to improve control of the spread of parasitic nematodes in this region.
